# Latent Correlations Between Longitudinal Systemic Complement Factors and Geographic Atrophy Growth Rate

**DOI:** 10.1167/iovs.67.12.69

**Published:** 2026-10-27

**Authors:** Talisa E. de Carlo Forest, Nathan C. Grove, Nicholas Cordaro, Yoga Advaith Veturi, Brandie D. Wagner, Niranjan Manoharan, Andres Lisker-Cervantes, Benjamin Bearce, Steve McNamara, Anne Lynch, Jayashree Kalpathy-Cramer, Alan G. Palestine, Marc Mathias, V. Michael Holers, Naresh Mandava

**Affiliations:** 1 Sue Anschutz-Rodgers Eye Center, University of Colorado Anschutz Medical Campus, Aurora, CO, United States; 2 Colorado School of Public Health, University of Colorado, Aurora, CO, United States; 3 Departments of Medicine and Immunology, University of Colorado School of Medicine, Aurora, CO, United States

**Keywords:** age-related macular degeneration, retinal imaging, complement, geographic atrophy

## Abstract

**Purpose:**

Investigate associations between square root-transformed (SQRT) geographic atrophy (GA) growth rate and longitudinal systemic complement levels in non-neovascular AMD.

**Methods:**

Prospective longitudinal cohort study of patients with GA in the University of Colorado AMD Registry enrolled from August 2014 to September 2022, with ≥1month of follow-up imaging through April 2023. Systemic complement factor level ratios (Bb/Factor B, Ba/Factor B, Factor H, Factor I, C4b/C4, C3a/C3, C3b/C3, sC5b-9/C5) were collected approximately annually. SQRT GA growth rate was quantified on fundus autofluorescence. Correlations between GA growth rate and longitudinal biomarker levels were evaluated using a Bayesian hierarchical joint model.

**Results:**

One hundred fifty-five eyes from 88 patients were included. Average SQRT GA growth rate was 0.27mm/year over a mean of 3.3 years and was positively associated with female sex. Ba/Factor B (correlation [credible interval {CrI}]:0.35, 95% CrI: 0.02 to 0.64), and Bb/Factor B (0.20, 95% CrI: −0.086 to 0.48) demonstrated posterior distributions largely above zero, consistent with a positive association. Posterior distributions for Factor I (−0.08, 95% CrI: −0.35 to 0.20) and C3a/C3 (−0.14, 95% CrI: −0.44 to 0.18) were centered below zero, whereas those for Factor H (0.09, 95% CrI: −0.20 to 0.37) and sC5b-9/C5 (0.09, 95% Crl: −0.19 to 0.37) were centered above zero.

**Conclusions:**

Systemic alternative pathway biomarkers demonstrated the strongest positive associations with GA growth rate. The pattern of associations in this exploratory analysis supports a hypothesis-generating framework in which differences in alternative pathway regulation may be associated with heterogeneity in GA progression. Future larger studies are needed for external validation of this hypothesis-generating work.

AMD is a leading cause of irreversible vision loss among elderly adults in developed countries, with the advanced forms responsible for the most severe visual morbidity.^1^ Geographic atrophy (GA), the advanced non-neovascular form of AMD, is a major cause of this irreversible vision loss, characterized by well demarcated areas of atrophy of the RPE, overlying photoreceptors, and underlying choriocapillaris.^2^ GA is a progressive disease with a highly variable progression. Annual growth rates range from approximately 0.53 to 2.6 mm^2^/year across different studies,^2^ ultimately leading to significant reductions in central vision and quality of life. The variability in GA progression rates has yet to be fully understood, posing challenges for prognostication and therapeutic development.

Growing evidence implicates dysregulation of the complement system, particularly the alternative pathway (AP), as a central driver of GA pathogenesis.^3^^–^^5^ In support, single nucleotide polymorphisms linked to AMD and GA have been identified in complement activation pathway and regulatory genes, including CFH, C2/CFB, CFI, C3, and C9.^6^^,^^7^ The complement system has three pathways that converge by activating C3, thereby producing the activation fragment C3a. Subsequently, the terminal pathway (TP) is activated, resulting in the formation of C5a and the membrane-attack complex, C5b-9.^8^^–^^10^ The classical pathway (CP) is initiated by C1q and the lectin pathway (LP) by mannose-binding lectin, ficolins, or collectins, and together these pathways lead to C4 activation. The AP is initiated by spontaneous hydrolysis of C3. Factor B then binds to C3b, and Factor D cleaves it into Ba and Bb. The complement system is also regulated by Factor H and Factor I, which inhibit the amplification loop by inactivating C3b.^11^

In 2023, the Food and Drug Administration approved two intravitreal complement inhibitors to slow the growth of GA, a C3 inhibitor pegcetacoplan and a C5 inhibitor avacincaptad pegol.^12^^,^^13^ Multiple drugs targeting the complement system locally and systemically have previously been, and continue to be, evaluated.^14^ Altogether, these events underscore the strong interest in targeting the complement system as a potential GA therapeutic strategy. However, crucially, there is limited data directly linking complement levels to the rate of GA progression, and identifying which pathway(s) are most involved.^15^ Most available studies indirectly addressed this by examining associations between GA growth and single nucleotide polymorphisms in complement-related genes.^6^^,^^16^^,^^17^ A better understanding of this relationship is essential for guiding future therapeutic strategies. The objective of the current study is to evaluate associations between square root-transformed (SQRT) GA growth rate in each eye and longitudinally measured systemic complement levels.

## Methods

This is a prospective longitudinal cohort study of patients with advanced non-neovascular form of AMD with GA enrolled in the University of Colorado AMD Registry from August 2014 to September 2022, and with follow-up through April 2023. The registry and this study were approved by the Colorado Multiple Institutional Review Board and conform to the Declaration of Helsinki. The recruitment process and informed consent of each patient in this AMD registry are described in prior publications.^5^^,^^18^ Informed consent was obtained from all study participants. The inclusion criteria for participants in the AMD registry are: patients 55 to 99 years of age, presence of AMD in at least one eye, and patient ability to give informed consent. The exclusion criteria included a history of concomitant retinal and uveitic disease, systemic cancer undergoing treatment, and autoimmune disease requiring immunosuppressive agents that can influence complement activity and are outlined in detail elsewhere.^19^ Multimodal imaging (MMI) and blood samples were obtained at enrollment and approximately every six months for MMI and annually for blood samples thereafter.

Patients were eligible for the present study if they had GA in at least one eye, no history of choroidal neovascularization and/or prior intravitreal injections, and at least two visits with MMI. Patients were categorized in the AMD Registry using the classification described by the Beckman Initiative for Macular Research Committee.^20^ The presence of GA was further defined using the Classification of Atrophy Meetings criteria for cRORA: (1) loss of outer retinal layers, (2) loss of RPE, and (3) choroidal hypertransmission of at least 250 µm in size in patients with AMD and no other retinal disease.^21^ Two vitreoretinal specialists confirmed the diagnosis at enrollment for accurate classification of AMD in the AMD Registry using MMI of color fundus photographs, fundus autofluorescence (FAF), and optical coherence tomography (OCT). The images and patient records were also reviewed by a third vitreoretinal specialist to ensure that no development of neovascular AMD or other concomitant exclusionary diseases occurred during the study period.

### Imaging Analysis

The MMI relevant to this study included FAF and OCT imaging. The Heidelberg Spectralis (Heidelberg Engineering Inc, Heidelberg, Germany) device was used to obtain a 30° or 55° FAF with automatic real-time function (ART) of 50 centered on the fovea. It was also used to obtain high-resolution a 49-line cube scan (6 mm bscans/30° scanning laser ophthalmoscopy/ART 9) centered on the fovea.

An artificial intelligence model (AI) for GA size detection on FAF images was created using the Medical Segment Anything Model,^22^ and evaluated on 342 expert annotated FAF images achieving an area correlation of *r* = 0.99 and *P* < 0.001 between model and expert area annotations. On an independent test set, the segmentation predictions from our algorithm showed a dice similarity score of 0.861 95% confidence interval [0.824:0.892] (n-194). To characterize the model's agreement between 840 AI-derived and expert-corrected GA measurements, before use in this article, we computed the concordance correlation coefficient as 0.989. This AI segmentation model was not used as a standalone grading tool, but rather to accelerate expert annotation. The AI model was run on FAF images at enrollment and last follow-up for each patient's eye(s) that had GA. A vitreoretinal specialist then reviewed and adjusted the automated FAF GA delineations, using spatially-registered infrared and OCT images from the same visit as additional guides as needed. SQRT GA growth rate was calculated for each eye with GA by taking the difference in SQRT GA size between the first and final visit and dividing by the time between visits. SQRT GA growth rate was used to better account for non-linear GA growth and to reduce dependence on baseline GA size.^23^

### Complement Measurements

The annual blood samples were drawn in an EDTA tube, and then spun using a cooled (40°C) centrifuge at 3000 revolutions per minute for 10 minutes to isolate plasma. Aliquots of plasma were stored in a −80°C freezer until they were pulled for analysis. Plasma samples from each patient were analyzed for complement factor levels and activation by ELISA (Quidel, San Diego, CA, USA) and Luminex immunoassays (MilliporeSigma, Burlington, MA, USA) as described in detail in our prior studies.^5^^,^^24^ In this study we measured complement levels and activation ratios (activation fragment/intact protein): Bb/factor B, Ba/factor B, Factor H, Factor I, C3a/C3, C3b/C3, C4b/C4, sC5b-9/C5. We chose to focus on the complement activation ratios rather than individual complement proteins and fragments because activation ratios are a better indication of complement activation.^25^^–^^27^ For each subject, plasma samples collected from up to six months before the first FAF image through six months after the final FAF image were eligible for inclusion.

### Statistical Analysis

Eye-level associations between SQRT GA growth rate and demographic variables were estimated using linear mixed effects models with random intercepts for subjects. Associations between longitudinally measured complement factors and factor ratios with eye-specific SQRT GA growth rate were estimated using a Bayesian hierarchical joint model. This approach jointly models longitudinal biomarker measurements within subjects and GA growth in each eye of a subject while accounting for within-subject correlation and unbalanced follow-up. Subject-specific random intercepts from all longitudinal sub-models were modeled jointly as following a multivariate normal distribution with an unstructured covariance matrix. Although SQRT GA growth rate was modeled at the eye level, latent random intercepts were estimated at the subject level using information from both eyes within a subject when available, allowing latent correlations between subject-specific biomarker profiles and SQRT GA growth rate to be estimated without averaging or otherwise pooling eye-specific SQRT GA growth rates within an individual prior to modeling.

Complement factors were corrected for batch effects and were log_2_-transformed and standardized (mean = 0, SD = 1); factor ratios were additionally adjusted for their denominator (parent protein). SQRT GA growth rate was standardized (mean = 0, SD = 1). Longitudinal complement factor trajectories were modeled using linear mixed-effects models with natural cubic splines for time and random intercepts for subjects. All available complement measurements for each subject were incorporated into longitudinal biomarker sub-models, so subjects with fewer measurements provided less information about their subject-specific biomarker profile, and their estimates were more strongly informed by the estimated population-level trajectories. To assess the effect of including subjects with one biomarker measurement, we performed a sensitivity analysis in which these subjects were excluded. Weakly informative priors were used to regularize estimation, particularly for the covariance structure, and all sub-models were adjusted for sex in the fixed effects.

Subject-specific random effects represent latent subject-specific deviations from the population-average biomarker trajectories and SQRT GA growth rate after accounting for the fixed effects. Associations between complement factors and SQRT GA growth rate were quantified through these latent correlations between subject-specific random effects across the joint model, representing associations between individual-level biomarker profiles and GA growth. Given the exploratory and comparative nature of this analysis, we focused on the pattern of associations across biomarkers, including consistency in direction and relative strength, rather than on whether individual correlations met conventional thresholds for statistical significance. Furthermore, because these correlations were estimated jointly as elements of a single random-effects correlation structure, rather than as a series of independent pairwise hypothesis tests, we did not dichotomize individual correlations according to whether their 95% credible intervals included zero. We therefore interpret estimates in the context of their direction, magnitude, precision, the overall pattern of associations across the joint structure, and coherence with biological expectations. R, version 4.5.2 was used for frequentist analysis and to generate plots, and Bayesian inference was performed using Stan, version 2.38.0, using the *cmdstanr* package, version 0.9.0. The Stan code used to fit the joint model can be accessed at https://github.com/ncgrove.

## Results

A total of 155 eyes from 88 patients were included in the analysis. In Table 1 we present the subject-level demographic characteristics of the cohort. The cohort was predominantly female (53%), and the mean (standard deviation [SD]) age at enrollment was 81 (7) years. The mean baseline GA area was 7.24 mm^2^ (range 0.066 mm^2^–35.53 mm^2^) and the average SQRT GA growth rate across all eyes was 0.27 mm/year (SD = 0.18, range 0–1.02) over a mean follow-up of 3.3 years (SD = 2.3, range 0.50 to 8.2). Three eyes had a growth rate of 0 over a follow-up period of 0.5, 1.5, and 1.5 years. Each patient contributed a mean of 2.3 complement measurements (SD = 1.32, range 1–5), with 33/88 patients having only one available measurement. Intraclass correlation coefficients for each complement measure ranged from 0.23 (C3a/C3) to 0.92 (C4b/C4), indicating varying degrees of within-subject variability across complement measures. In Table 2, we present the cohort's eye-level demographic characteristics. SQRT GA growth rate was not associated with demographic characteristics except for a positive association with female sex (*P* = 0.01).

**Table 1.** Characteristics of the Cohort

Characteristic (*N* = 88)	
Sex	
Male	41 (47%)
Female	47 (53%)
Age (years)	
Mean (SD)	81 (7)
Min, Max	64, 98
Race/Ethnicity	
White	81 (92%)
Black	1 (1.1%)
Hispanic	6 (6.8%)
Other/Unknown	0 (0%)
Smoking	
Never	37 (42%)
Current	4 (4.5%)
Former	47 (53%)
Family history of AMD	
None	49 (56%)
Parent	22 (25%)
Sibling	7 (8.0%)
Uncertain	10 (11%)
Number of biomarker samples	
Mean (SD)	2.32 (1.32)
Min, Max	1.00, 5.00
Eye-level SQRT GA Growth Rate (mm/yr) (*n* = 155 eyes)	
Mean (SD)	0.2736 (0.1789)
Min, Max	0.0000, 1.0213
Eye-Level Baseline GA Area (mm^2^) (*n* = 155 eyes)	
Mean (SD)	7.2445 (8.4529)
Min, Max	0.0655, 35.5307

**Table 2.** Eye-Level Associations of Cohort Characteristics With Square Root Geographic Atrophy Growth Rate

Characteristic	*N*	**β**	95% CI	*P* Value
Sex	155			
Male		—	—	
Female		0.09	0.02, 0.15	**0.010**
Age (years)	155	0.00	0.00, 0.01	>0.9
Race/Ethnicity	155			
White		—	—	
Black		−0.19	−0.51, 0.12	0.2
Hispanic		0.04	−0.10, 0.17	0.6
Smoking	155			
Never		—	—	
Current		0.02	−0.15, 0.19	0.8
Former		−0.06	−0.13, 0.01	0.11
Family history of AMD	155			
None		—	—	
Parent		−0.01	−0.09, 0.08	0.9
Sibling		0.00	−0.13, 0.13	>0.9
Uncertain		0.00	−0.12, 0.11	>0.9

CI, confidence interval.

Associations estimated using linear mixed effects models with random intercepts for subjects.

Bolded *P* values indicate statistically significant values.

In Table 3, we summarize the posterior distributions of the correlations between longitudinal complement measures and SQRT GA growth rate. Complement ratios C3b/C3 and C4b/C4 had posterior distributions centered near zero, indicating limited evidence for association. The AP regulator Factor I (posterior mean = −0.076, 95% Credible Interval (95% CrI): −0.347 to 0.198) and C3a/C3 (−0.138, 95% CrI: −0.439 to 0.182) had posterior distributions centered below zero, suggesting potential negative associations with SQRT GA growth rate. The AP regulator Factor H (0.092, 95% CrI: −0.195 to 0.373) and TP ratio sC5b-9/C5 (0.094, 95% Crl: −0.190 to 0.373) had posterior distributions centered above zero, suggesting possible positive associations with SQRT GA growth rate. The Complement AP ratios Ba/Factor B (0.350, 95% CrI: 0.021 to 0.644), and Bb/Factor B (0.203, 95% CrI: −0.086 to 0.475) demonstrated the largest magnitude correlations with posterior distributions largely above zero, consistent with a positive association with SQRT GA growth rate.

**Table 3.** Summary of Posterior Distributions of Correlations Between Complement Factors and Square Root Transformed Geographic Atrophy Growth Rate

Variable	Mean	q2.5	q97.5
Ba/FactorB	0.350	0.021	0.644
Bb/FactorB	0.203	−0.086	0.475
C3a/C3	−0.138	−0.439	0.182
C3b/C3	0.038	−0.397	0.446
C4b/C4	−0.001	−0.278	0.272
sC5b_9/C5	0.094	−0.190	0.373
FactorH	0.092	−0.195	0.373
FactorI	−0.076	−0.347	0.198

q, quantile.

Correlations were estimated using a Bayesian hierarchical joint model of complement factors and complement factor ratios (activation fragment/parent protein) and square root-transformed geographic atrophy growth rate adjusting for sex in all sub-models.

The posterior distributions of the complete joint random effects correlation matrix are shown in Figure 1. Specifically, there was evidence that Factors H and I were negatively associated with Bb/Factor B although positively associated with C3a/C3, C4b/C4, and Ba/Factor B. Additionally C4b/C4 demonstrated a potential association with C3a/C3, but not C3b/C3, either Factor B ratio, or sC5b-9/C5.

**Figure 1.**
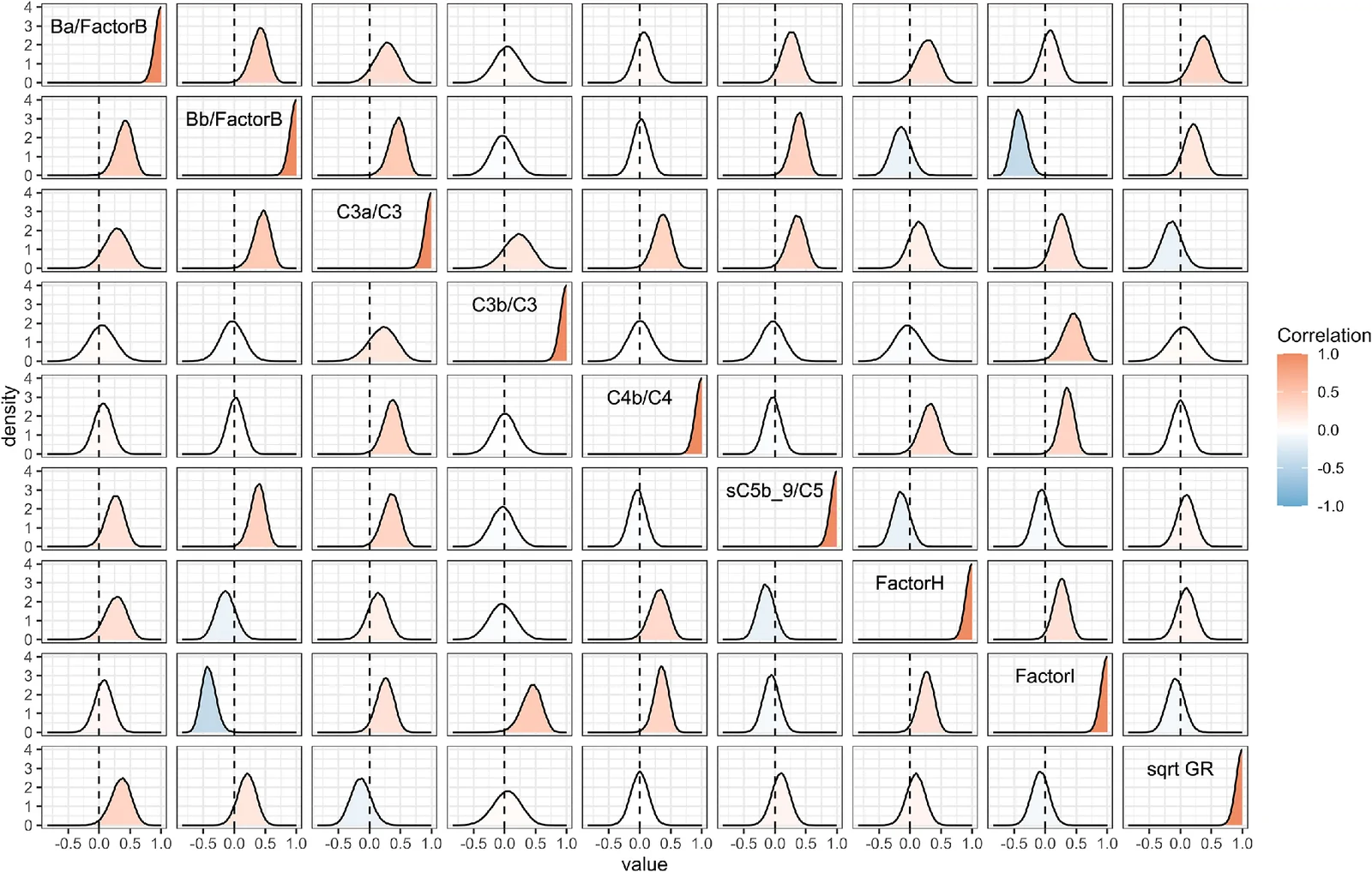
Posterior distribution of joint random effects correlation matrix for all complement factors and square root-transformed GA growth rate. Correlations were estimated using a Bayesian hierarchical joint model of complement factors and complement factor ratios (activation fragment/parent protein) and SQRT geographic atrophy growth rate (SQRT GR). Each panel displays the posterior distribution of the correlation between a pair of subject-specific random effects. *Diagonal panels* identify model components, and *off-diagonal panels* show pairwise correlations. The *dashed vertical line* indicates zero correlation. Shading reflects the direction and magnitude of the posterior mean correlation (*red*, positive; *blue*, negative), and the width of each distribution reflects estimation uncertainty. Correlations on the bottom row and rightmost column represent subject-specific associations between complement factor levels and geographic atrophy growth rate after adjustment for sex.

In a sensitivity analysis excluding the 33 subjects with only one complement measurement, the estimated correlations for Ba/Factor B and Bb/Factor B remained positive and of similar magnitude to the primary analysis (Supplementary Table S1). Furthermore, the correlations for sC5b/C5 remained positive with a much stronger magnitude than the primary analysis and those for Factors H and I shifted minimally in magnitude.

## Discussion

In this study, we demonstrate that longitudinally measured systemic complement activity may be associated with GA progression. Specifically, our findings support the theory that elevated systemic AP complement activity may be associated with GA growth rate and support further investigation in larger cohorts. This suggests that systemic complement biomarkers may reflect or contribute to retinal disease activity in GA. We also found an association between female sex and faster GA growth. Sex was included as a covariate in the complement models given both this association and prior evidence of sex-related differences in complement biology. Although our study was not designed to investigate sex-specific mechanisms, this finding is notable given growing evidence that sex can influence immune and inflammatory responses, including components of the complement system.^24^^,^^28^^,^^29^ Whether sex-related differences in complement regulation contribute to variation in GA progression remains unclear and warrants investigation in larger studies specifically designed to evaluate these relationships.

Longitudinal measurements of activation ratios Ba/Factor B and Bb/Factor B were positively correlated with SQRT GA growth rate, suggesting that overactivation of the AP is seen in faster GA progression. Similarly, we found modest evidence that SQRT GA growth rate may be correlated with C3a/C3 and Factor I, and positively correlated with C5b-9/C5 and Factor H. Interpretation of these associations is more complex. Importantly, these results reflect subject-specific relative differences within our GA study cohort and do not imply, for example, that C3a/C3 activity is absolutely reduced in faster progressors, but rather that C3a/C3 could be lower in faster progressors relative to those with slower progression.

To better digest and contextualize our results, we evaluated the relationships between the complement factors and ratios using the posterior distributions of the complete joint random effects correlation matrix (Fig. 1), developing speculative models for future validation. In doing so, our results suggest a possible conceptional framework that warrant further investigation of two putative complement activation states that differentially influence progression of GA in AMD: (1) an AP amplification–dominant state associated with faster GA progression, and (2) a regulated complement turnover state, partly involving CP activity, associated with slower GA progression (Fig. 2).

**Figure 2.**
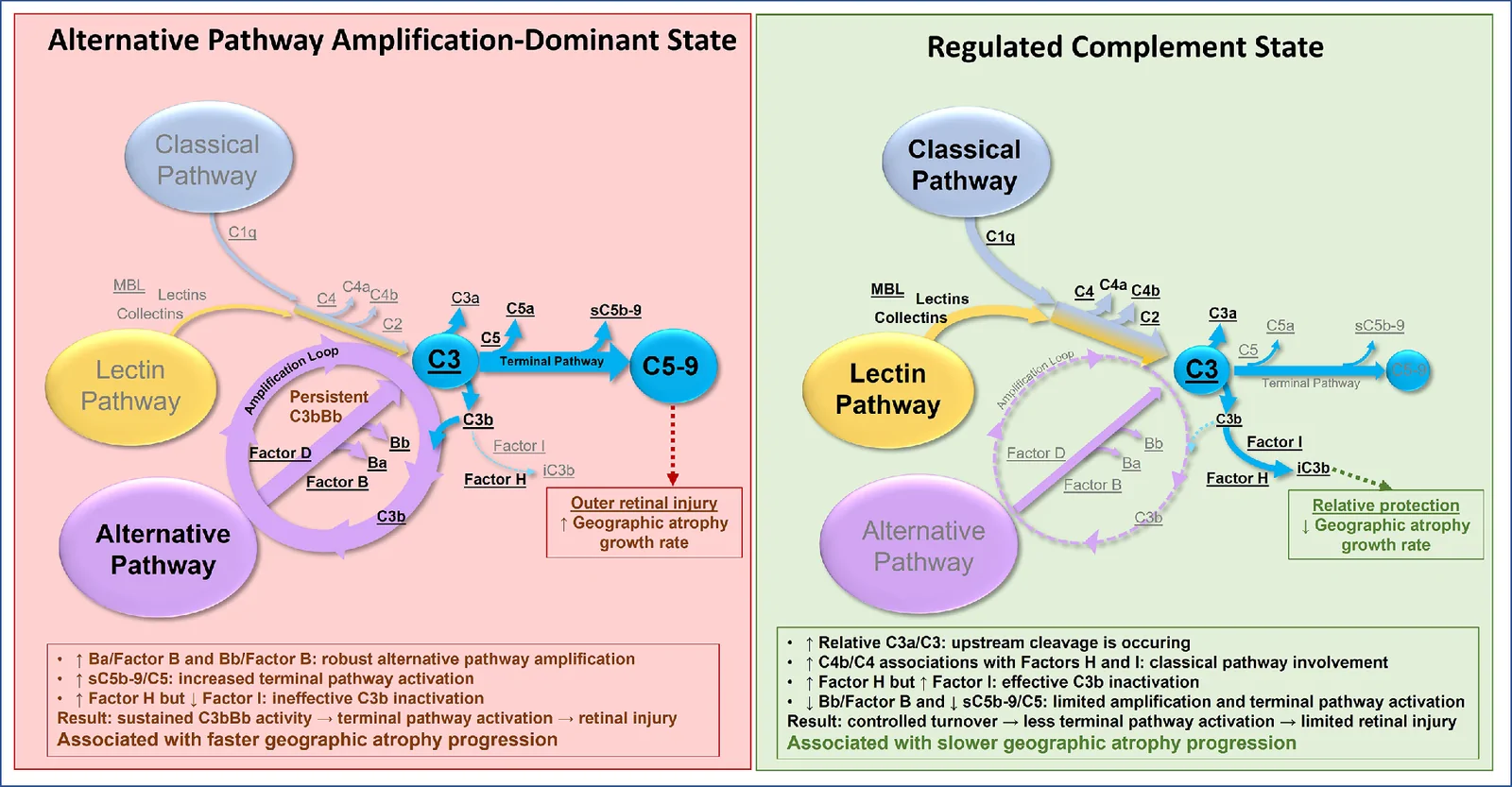
Proposed complement pathway states. We propose a hypothesis-generating conceptual framework consisting of two putative complement activation states that we theorize could differentially influence progression of GA in AMD: a proposed AP amplification–dominant state associated with faster GA progression (*left*), and a regulated complement turnover state, partly involving CP activity, that we postulate could be associated with slower GA progression (*right*). These biological interpretations should be viewed as an illustration of exploratory hypotheses requiring further validation prior.

In the first hypothesized state, faster GA growth is characterized by increased AP amplification and downstream TP activation. This is supported by modest positive associations between SQRT GA growth rate and Ba/Factor B, Bb/Factor B, and sC5b-9/C5. These associations are consistent with a hypothesis in which C3b successfully enters and sustains the AP C3 convertase (C3bBb) amplification loop, leading to increased TP activity and retinal tissue injury. Although we found that Factor H may be elevated in faster GA progression, consistent with a compensatory regulatory response, we found that Factor I was possibly decreased in faster progressors. If these associations reflect underlying biology, this could be consistent with less effective cofactor-mediated C3b inactivation in the absence of adequate Factor I. In addition, Factor I showed a strongly negative association with Bb/factor B, a key marker of AP amplification, supporting a model in which reduced C3b inactivation could permit persistence of AP C3 convertase activity and downstream TP activation.

In contrast, the second hypothesized state is characterized by slower GA progression despite evidence of ongoing complement cleavage. Here, C3a/C3 was somewhat negatively associated with the GA growth rate, suggesting that C3 activation occurs but does not translate into stable AP C3 convertase formation or TP activation. Rather, C3a/C3 was more positively associated with Factor I and Factor H, potentially indicating efficient regulation of C3b through Factor H–mediated binding and Factor I–mediated irreversible inactivation before entry into the AP amplification loop. The positive association of C4b/C4 with C3a/C3, Factor H, and Factor I, but not with C3b/C3, Ba/factor B, Bb/factor B, or sC5b-9/C5, suggests that CP activation may contribute to the upstream C3 cleavage without progression to AP amplification. Congruently, while both Factor H and Factor I were positively associated with upstream cleavage markers (C3a/C3 and C4b/C4), they were negatively associated with Bb/factor B.

Taken together, these findings support a conceptual framework in which GA progression may not only be determined by the overall magnitude of complement activation, but also by the extent to which activated C3 escapes Factor H– and I–mediated regulation thereby entering the amplification loop that drives TP-mediated retinal injury. Because several estimated associations were accompanied by moderate posterior uncertainty, the biological interpretations discussed above should be viewed as exploratory hypotheses rather than evidence of established complement phenotypes. These hypothesized states require validation in independent cohorts before being interpreted as biologically established phenotypes.

To minimize potential confounding from neovascular disease activity and concomitant anti-vascular endothelial growth factor treatment, we restricted our analysis to eyes with GA in the absence of concurrent neovascular AMD. Further, segmentation of atrophy in the setting of neovascular AMD can be challenging. However, recent real-world studies have suggested that eyes with both GA and neovascular AMD may represent an important biologically distinct subgroup, with evidence of meaningful and potentially differential responses to local complement inhibition.^30^^,^^31^ Future studies should evaluate whether relationships between systemic complement biomarkers and GA progression differ in eyes with concurrent neovascular AMD. Such investigations may provide further insight into biological heterogeneity within advanced AMD.

Prior complement-related genetic studies have inconsistently detected significant associations with GA growth rates, possibly in part because genetics represent only one of several factors influencing complement levels in the body.^6^^,^^16^^,^^17^^,^^32^ Our current work evaluates the association between systemic complement and GA growth rate more directly while supporting prior work that showed associations between GA growth rate and complement genetic variants associated with AP regulation such as CFH, CFB, and CFI.^6^^,^^32^ Interestingly, our results differ from the only other comparable study we are aware of, which found no significant associations between GA growth rate and complement levels in the aqueous humor or plasma.^15^ This study differed from ours in several key ways, including having a limited sample size of 26 patients, no consideration of interactions among complement factors, and the use of GA growth rate without adjusting for baseline GA size or square-root transformation. The prior study relied on FAF-based measurements of GA, whereas our study used spatially-registered OCT and infrared images to guide final FAF annotations, which could improve accuracy, especially subfoveally or in borderline lesions on FAF. Additionally, the analysis was limited to a single baseline measurement of systemic Factor B, C3, C4 levels, fragments, and especially activation ratios, without including key regulators, which were key in our study. In contrast, the use of longitudinal complement ratio and regulator level measurements in our study allows for the assessment of associations between GA growth rate and sustained complement activity, while accounting for correlations among complement factors. However, our study did not include measurements of local complement levels, unlike the other study. Because systemic and intraocular complement levels may differ, our findings should not be extrapolated to local complement activity.

Strengths of our study include the prospective design, inclusion of longitudinal complement activation ratio data, exploration of complement factor relationships, the use of advanced MMI measurement of GA growth, and a joint modeling approach. This study was limited by a single GA outcome, SQRT GA growth rate. While our approach sacrifices information about within-eye temporal variation in GA growth, this outcome was derived before joint modeling to avoid inclusion of additional subject- and eye-specific random effects terms in the growth sub-model, which would have increased both the model dimensionality and number of parameters in the shared random-effects correlation matrix. Future studies with larger sample sizes should investigate joint models incorporating longitudinal GA growth trajectories (including temporospatial growth modeling metrics), baseline characteristics (GA size, focality, foveal-involvement) and systemic complement measures. Although we had more than three times as many subjects as the most comparable study, we were still limited by a modest sample size.^15^ Additionally, although our study was distinguished by longitudinal complement biomarker levels, it was still limited by relatively sparse duplicate measures in some individuals. Finally, systemic complement levels were measured at the patient level, whereas GA progression is by nature eye-specific; this limited our ability to capture eye-level differences in growth and complement associations. Future larger studies are needed for external validation of this hypothesis-generating work and should potentially integrate genetics and local (ocular) complement data.

Altogether, this study advances the current understanding of the relationship between longitudinal systemic complement activation and GA growth rate. Our work generates a hypothesis-generating conceptual framework for a potential key role of Factor I–mediated regulation in GA progression. Although this hypothesis requires further investigation, the overall findings reinforce continued investigation of systemic complement biomarkers as potential correlates of disease progression. They also support further investigation of systemic complement suppression for GA, particularly those targeting modulation of the AP.

## Supplementary Material

Supplement 1
