# Supplementary material for: Latent Correlations Between Longitudinal Systemic Complement Factors and Geographic Atrophy Growth Rate
Source: Invest Ophthalmol Vis Sci. 2026 Oct 27;67(12):69. doi: 10.1167/iovs.67.12.69 (PMC13722971; doi:10.1167/iovs.67.12.69)
Supplement: Supplementary file 1 [file iovs-67-12-69_s001.pdf]

Supplement Table 1. Summary of Posterior Distributions of Correlations Between Complement Factors and Square Root Transformed Geographic Atrophy Growth Rate (all sub-models adjusted for sex) From a Sensitivity Analysis in Which All Subjects Have at Least Two Longitudinal Biomarker Samples

Variable	Mean	q2.5	q97.5
**Ba/FactorB**	0.272	-0.072	0.582
**Bb/FactorB**	0.282	-0.025	0.563
**C3a/C3**	0.001	-0.356	0.364
**C3b/C3**	0.057	-0.365	0.444
**C4b/C4**	-0.131	-0.438	0.190
**sC5b_9/C5**	0.243	-0.064	0.525
**FactorH**	0.044	-0.278	0.354
**FactorI**	-0.187	-0.466	0.113
